# Editorial: Theory and practice of soccer psychology

**DOI:** 10.3389/fpsyg.2026.1782541

**Published:** 2026-01-26

**Authors:** Erkut Konter, Garry Kuan, Itay Basevitch, Erwin Apitzsch

**Affiliations:** 1 Istanbul Gelisim University, Faculty of Sport Sciences, Istanbul, Türkiye; 2 Universiti Sains Malaysia, George Town, Malaysia; 3 Barry University, Miami Shores, FL, United States; 4 Lunds Universitet, Lund, Sweden

**Keywords:** applied soccer psychology, diversity, soccer coaching psychology, soccer health psychology, soccer performance psychology, soccer psychological consultancy, soccer psychological skills

Soccer is the most widely played and followed sport worldwide (Reilly and Williams, 2023). Over the past decade, soccer psychology has emerged as a rapidly expanding interdisciplinary field encompassing diverse and multifaceted domains (Konter and Loughead, 2019). Moreover, sport psychology consultation is more widely accepted and has grown immensely in the twenty-first century (Aoyagi et al., 2012). Therefore, the study of soccer psychology incorporates a broad array of topics from psychology and sport sciences (Dixson and Mitchell, 2020; Konter and Loughead, 2019).

Psychological preparation in general, and coping and mental strategies in particular are increasingly being recognized as a critical determinant of success, representing a cutting-edge component in modern soccer. Many soccer teams now routinely provide sport psychology services for their athletes' psychological preparation, health and performance enhancement (Strudwick, 2016). The demands placed on sport psychologists and consultants continue to rise within soccer clubs, Olympic teams, and national squads. Therefore, the latest soccer specific evidence-based knowledge is required (Konter and Loughead, 2019).

This e-book features chapters authored by seasoned researchers and practitioners with extensive experience in soccer psychology. We are pleased to announce that our new e-book, titled *Theory and practice of soccer psychology*, is now published by Frontiers in Psychology. It comprises eight recent research articles, authored by international experts, covering a broad spectrum of topical areas, including mental health, cognitive development, probabilistic modeling of penalty kicks, temperament traits, gender participation, coach-athlete relationships, burnout, and athlete satisfaction. The select papers include various research designs, gender, age groups, and experience level of soccer players. The objective of this e-book is to provide the most up-to-date knowledge and cover advances in soccer psychology for sport psychology consultants, coaches, scouts, managers, researchers and students in the sport sciences.

Andrade et al. conducted a systematic review examining the psychological impacts of the COVID-19 pandemic on elite soccer athletes. The investigation of the pandemic's impact on soccer players psychological processes, wellbeing and mental health is essential given the unique challenges in elite soccer environments, including training disruptions and competition postponements. The authors discuss and note that the knowledge gained from the review will allow the development of specific strategies to preserve mental health and performance of elite athletes in similar situations and in general, contributing to effective interventions with both short and long-term benefits.

Mao F., Li Z. et al. in their opinion paper promote integrating in physical education settings basic soccer skills training sessions to stimulate cognitive promotion for children and adolescents. They highlight that intermittent running at various levels of intensities associated with jumps, sprints, accelerations, and changes of direction in soccer play an important role for physical health, fitness and cognitive functions including decision-making, perception, observation, and action for children and adolescents. In addition, soccer offers children and adolescents affordances to stimulate cognitive development.

Campo et al. utilized a case study approach, examined the effect of an 8 week on-field training program for a youth goalkeeper where probabilistic information about soccer penalty kickers tendencies were provided. The study was based on the notion that in a penalty shot situation in soccer there is direct interaction between the shooter and goalkeeper where both search for relevant information as a means to achieve their respective performance goals. This paper is especially important as penalty kicks can determine the final outcomes, of games, especially in high stake situations such as during large tournaments or in crucial elimination matches. Based on the findings from the case study, the authors propose to expand the training with probabilistic information of the kickers to elite goal keepers.

Bojkowski and Tomczak examined in their study the link between temperament structures and effectiveness of individual play in soccer. The authors used Regulatory Theory of Temperament to guide their research. The theory distinguishes two traits involving the time course of responses and four relating to how energy is distributed and stored. The theory enables the presentation of entire structures of temperament traits, and it is relevant for assessing an individual's ability to process stimulation and influence their performance during competitions. Specifically, these structures were examined in relation to the offensive, defensive, and comprehensive effectiveness of individual soccer players' actions.

Zeng and He investigated adolescent participation in soccer. The researchers examined the intersection of gender and sports participation in adolescence, focusing on traditionally male-dominated sports like soccer. Their research was guided by the Theory of Planned Behavior (TPB), and they used structural equation modeling to examine gender-differentiated patterns and factors influencing adolescent participation in soccer. The researchers collected a large sample of participants (*n* = 1.147). This research highlights the complex role of gender in soccer participation and provides strategic insights for increasing girls' involvement in sport. The findings set the stage for future research on enhancing girls' participation in male dominant sports such as soccer.

Liu and Li examined the correlation between coach-athlete relationship and burnout among college soccer players considering the mediating role of training satisfaction in their research. Athlete burnout leads to decreases in performance, success, and enjoyment and increase of health problems. Burnout decreases athletes' sports performance and reduces their passion for continuing their athletic career development often leading to athletes dropping out of athletic participation. The study examined the association of coach-athlete relationships, training satisfaction, and athlete burnout. In addition, the authors explored training satisfaction as a mediating role of the effect of coach-athlete relationship on athlete burnout among college soccer players (*n* = 218) in Chinese higher education institutions. Based on the findings the authors indicated the importance of communication to improve the coach-athlete relationship and enhance training satisfaction by building team culture to reduce burnout among collegiate soccer players.

Mao F., Yin A. et al. conducted a meta-analysis to examine the effects of soccer training on cognitive performance in children and adolescents. The authors indicated that the cognitive development of children and adolescents is crucial for their academic success and overall wellbeing. Physical activity has been linked to improved cognitive performance, but the specific effects of soccer training on cognitive function in this population remain unclear. Therefore, the authors conducted a meta-analysis and comprehensively evaluated the impact of soccer training on cognitive performance. Significant results of the effects of soccer training on cognition in children and adolescents (*n* = 1,574 children and 94 adolescents, *p* < 0.05) were found. The findings suggested that soccer training positively influences cognitive performance in children and adolescents particularly in attention, inhibitory control, and working memory. The authors concluded that environmental enrichment, cardiovascular fitness, and cognitive component skills help elucidate the underlying mechanisms of these effects. These results indicate that the inclusion of soccer in educational programs is important to foster cognitive development.

Li et al. investigated the neuromotor mechanisms of successful soccer penalty kicking using EEG analyses. Understanding the neuromotor processes underlying successful and unsuccessful performance in lower limb movements, such as soccer kicking, is essential for players. Therefore, lower activation of the prefrontal and central cortices associated with motor programming (such as motor planning and motor control) is crucial for psychomotor performance in difficult tasks such as soccer penalty kicks. To address this issue, the authors investigated neuromotor processes in skilled soccer players performing penalty kicks under difficult conditions (*n* = 10, 30 kicks each, 40–60% success rate). Findings support the model of attention allocation and the psychomotor efficiency hypothesis. Overall, this study highlights the critical role of motor planning and control in successful athletic performance such as soccer penalty kicks.

This e-book offers essential readings grounded in cutting-edge literature for students, researchers, and professionals interested in the field of soccer psychology. Covering a diverse array of topics, it aims to advance understanding and practice within the area of soccer psychology.
